# New horizons in the treatment of psoriasis: Modulation of gut microbiome

**DOI:** 10.1016/j.heliyon.2025.e41672

**Published:** 2025-01-03

**Authors:** Mojtaba Memariani, Hamed Memariani

**Affiliations:** a Department of Mycobacteriology and Pulmonary Research, Pasteur Institute of Iran, Tehran, Iran; b Microbiology Research Center (MRC), Pasteur Institute of Iran, Tehran, Iran; c Department of Medical Microbiology, Tehran University of Medical Sciences, Tehran, Iran

**Keywords:** Psoriasis, Dysbiosis, Microbiota, Probiotics

## Abstract

The last decennia have witnessed spectacular advances in our knowledge about the influence of the gut microbiome on the development of a wide swathe of diseases that extend beyond the digestive tract, including skin diseases like psoriasis, atopic dermatitis, acne vulgaris, rosacea, alopecia areata, and hidradenitis suppurativa. The novel concept of the gut-skin axis delves into how skin diseases and the microbiome interact through inflammatory mediators, metabolites, and the intestinal barrier. Elucidating the effects of the gut microbiome on skin health could provide new opportunities for developing innovative treatments for dermatological diseases. Psoriasis is a complex disease with multiple factors contributing to its development, such as diet, lifestyle, genetic predisposition, and the microbiome. This paper has a dual purpose. First, we outline the current knowledge on the unique gut microbiota patterns implicated in the pathogenesis of psoriasis. Second, and of equal importance, we briefly discuss the reciprocal impact of psoriasis treatment and gut microbiome. In addition, this review explores potential therapeutic targets based on microbial interventions, which hold promise for providing new treatment options for psoriasis.

## 1 Introduction

The gut-skin axis describes the intricate relationship where the gut can influence skin health owing to its immunological and metabolic properties [1]. The gut ecosystem offers specific niches that supply nutrients and ideal growth conditions for the gut microbiome, which, in turn, executes a variety of functions essential for sustaining homeostasis in the body. The gut microbiome is essential for maintaining the health and homeostasis of the host, mediating inflammatory processes, and modulating immune responses through a nuanced balance of beneficial and harmful bacteria. Nevertheless, various factors such as lifestyle choices, dietary habits, bacterial infections, antibiotic use, surgical procedures, frailty, and inflammation can alter the composition of the gut microbiome. An imbalance in the gut microbiome results in microbial dysbiosis. This dysbiotic state is characterized by a decrease in the diversity of bacterial species and a reduction in the abundance of beneficial bacteria [2]. Recent years have witnessed a rise in research focusing on the role of the gut microbiome in human health, immune function, and disease development. Growing evidence suggests that gut dysbiosis is often present in common inflammatory skin diseases such as atopic dermatitis, rosacea, acne vulgaris, and psoriasis [3].

Psoriasis is a chronic inflammatory skin condition with a complex etiology that is non-contagious. It is relatively common in the general population and can affect people of all ages in all countries, regardless of ethnic origin [4]. Common clinical manifestations include well-defined, red, itchy patches with flaky, silver-white scales. These patches have the potential to extend over significant portions of the skin as they coalesce. The scalp, trunk, and extensor surfaces of the limbs are commonly involved regions [5]. In psoriasis, T cell activity, specifically T helper 17 (Th17) cells, is implicated in the disease process through the secretion of proinflammatory cytokines interleukin-17A (IL-17A) and IL-22. Such cytokines are responsible for the proliferation of keratinocytes and the activation of synoviocytes. The disease is marked by excessive proliferation of epidermal keratinocytes, dysregulated differentiation of these cells, increased vascularization, and inflammation of the dermis and epidermis [6].

Psoriasis may also impact the joints, leading to psoriatic arthritis. Several coexisting conditions have been reported, suggesting that psoriasis is a systemic condition rather than solely a skin disease. Psoriasis may elevate the likelihood of developing metabolic syndrome, diabetes, Crohn's disease, ulcerative colitis, obesity, certain cancers, and cardiovascular diseases [7]. The relationship between gut microbiota and the immune system is well recognized. The gut microbiome contributes significantly to the development and regulation of immune homeostasis by interacting with both innate and adaptive immune components. Disturbances in the gut microbiome or changes in the host-microbiome interfaces can initiate an immune response, heightening the risk of pathogenic invasion. In psoriatic patients, alterations in the gut microbiome can lead to both systemic and localized inflammation, which may enhance susceptibility to various systemic diseases. In genetically susceptible individuals, dysbiosis in gut microbiome can result in regulatory T cell (Treg) dysfunction and the activation of Th17 and innate lymphoid cells 3 (ILC3), which may contribute to the development of psoriasis and other systemic diseases [8]. The nutritional status of psoriatic patients can also affect the composition of gut microbiome, which in turn influences the progression of the disease and associated comorbidities [9]. For instance, a decrease in dietary fiber may result in a decline of short-chain fatty acids (SCFAs)-producing bacterial phyla in the gut microbiome. This alteration can activate dendritic cells in the gut, causing them to secrete IL-23, which may further stimulate the differentiation of unstable FOXP3-positive Tregs into IL-17-producing cells [8].

Given the implication of gut dysbiosis in the physiopathology of inflammatory and autoimmune diseases, increasing emphasis is now being put on maintaining intestinal microecological balance as a novel target for the prevention and treatment of psoriasis [10]. This brief review strives to outline the findings concerning alterations in the gut microbiome of psoriasis patients, as well as the potential influence of psoriasis medications on the intestinal microbiome. Furthermore, it attempts to explore potential therapeutic targets based on the manipulation of microbial communities, which may inaugurate new avenues for the treatment of psoriasis. We tried to present the up-to-date insights on the correlation between the intestinal microbiome and psoriasis in a manner that is easily comprehensible for scholars across different disciplines, from microbiology to dermatology.

## 2 Gut microbiome and human health

There is a wealth of evidence supporting the existence of the gut-skin axis and the inflammatory effects caused by an imbalance in the gut microbiome. Comprising a diverse array of bacterial species, the intestinal microbiome also includes protozoa, viruses, and fungi that reside mainly in the lower gut, playing a quintessential role in maintaining a symbiotic relationship with the host [3]. The intricate connection between the intestinal microbiota and the host is maintained through a complex network of interactions involving metabolic, immune, and neuroendocrine crosstalk. The gut microbiota assists in the digestive processes, produces vitamins, and controls the permeability of the intestinal wall [11]. When considering the wider picture, it becomes apparent that the gut microbiota and the endocrine system bear many similarities in their capacity to impact distant organs and systems [12].

The gut microbiota is essential for the fermentation of unabsorbed starch and soluble dietary fiber, resulting in the production of SCFAs such as butyrate, propionate, acetate, and pentanoate [13]. These SCFAs function as energy sources for the host. SCFAs, particularly butyrate, suppress immune responses by hindering the growth, movement, attachment, and cytokine release of inflammatory cells [14]. Furthermore, SCFAs block histone deacetylase and deactivate nuclear factor kappa B (NF-κB) signaling pathways to modulate the activation and apoptosis of immune cells. SCFAs also promote the activation of Tregs and induce the differentiation of B cells into plasma cells to produce specific immunoglobulin A (IgA) [15]. Apart from SCFAs, vitamins synthesized by gut microbiota are crucial for enhancing the metabolism of both microbes and the host. For instance, vitamin K2 is critical in decreasing vascular calcification, raising high-density lipoprotein (HDL), and lowering cholesterol levels, thereby reducing the likelihood of cardiovascular diseases such as atherosclerosis and coronary heart disease [16].

The transformation of primary bile acids by the gut microbiota can regulate host lipid and glucose metabolism. An intriguing aspect of bile acids is their anti-microbial properties. In addition, microbiota-derived secondary bile acids have the ability to enter the circulation and influence glucose homeostasis by interacting with receptors similar to those of the primary compounds [17]. The anti-microbial effects of secondary bile acids result in the alteration of microbial cell membrane integrity, causing the leakage of intracellular contents and thereby impeding the growth of bile acid-intolerant microorganisms. These anti-microbial properties are instrumental in shaping the gut microbiota composition and shielding the host from a range of infectious pathogens [18]. Microbial metabolic processes involve the conversion of choline, crucial for lipid metabolism, into trimethylamine. Following synthesis by intestinal bacteria, trimethylamine can be metabolized in the liver to trimethylamine-*N*-oxide (TMAO). Elevated levels of TMAO in the blood may contribute to the development of cardiovascular disease and stroke [19].

**Fig. 1:**
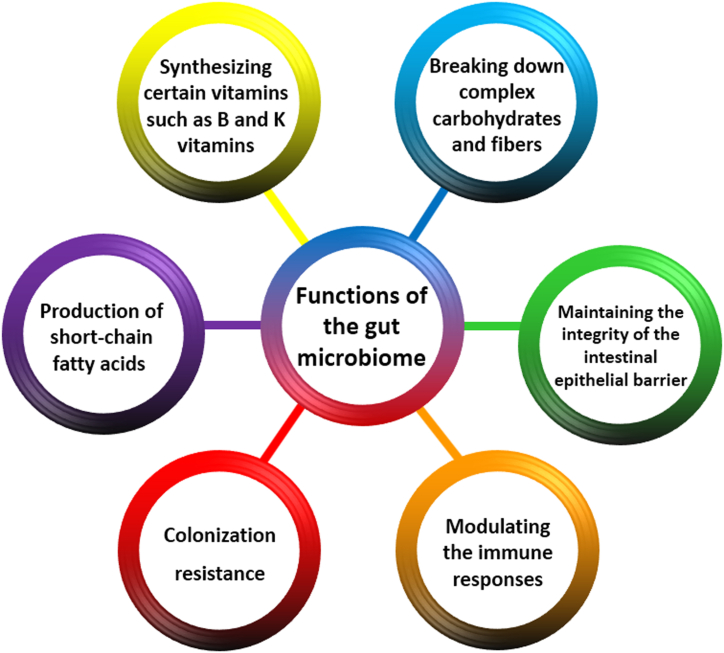
Functions of the gut microbiome encompass synthesizing specific vitamins, degrading complex carbohydrates and fibers, maintaining intestinal barrier integrity, producing short-chain fatty acids, bolstering colonization resistance, and modulating the immune responses.

Another vital role of the human microbiota is to provide colonization resistance, which helps protect the host from pathogenic invaders and prevents the overgrowth of pathobionts [20]. Although the molecular basis of colonization resistance is not fully comprehended, the proposed mechanisms of action can be attributed to direct competition between human microbiota and pathogens for shared resources and habitats, as well as the enhancement of the host's defense mechanisms by human microbiota to prevent pathogen invasion. The predominant non-pathogenic members of the gut microbiome are essential for occupying their specific niches and suppressing the growth and colonization of pathogenic microorganisms [21]. Nevertheless, disruptions in the gut microbiome can cause a decline in dominant microbiota members, reducing the overall ability to resist colonization. This circumstance affords a chance for opportunistic pathogenic strains to encroach upon or colonize vacant niches, thereby culminating in infections [22]. The different functions of the gut microbiome are illustrated in Fig. 1.

## 3 Gut microbiome alterations in psoriasis

Emerging data indicate that gut dysbiosis may be associated with the pathogenesis of numerous diseases including, but certainly not limited to, inflammatory bowel disease (IBD), type 2 diabetes, ulcerative colitis, Crohn's disease, asthma, obesity, metabolic syndrome, cardiovascular diseases, autoimmune disorders, and neurodevelopmental conditions [23]. Therefore, the scientific community is now investigating the correlation between pathological conditions and dysbiosis to determine if it is bidirectional or causal, how genetics and environmental stimuli affect its composition, and whether the gut microbiota can impact susceptibility to diseases and response to particular treatments [24]. Gut microbiota and their metabolites are instrumental in supporting host health through the process of food digestion and the maintenance of immune system balance. Within the gut microbiome, there exists a diverse range of bacterial phyla, predominantly composed of *Firmicutes*, *Bacteroidetes*, *Actinobacteria*, *Proteobacteria*, *Fusobacteria*, and *Verrucomicrobia*. This complex ecosystem also includes viruses, fungi, protozoa, and Archaea, all of which foster a symbiotic connection with the host. The nature of this relationship is intricately influenced by the age, genetic makeup, dietary patterns, and external environmental factors [25,26].

Those with psoriasis experience a reduced diversity of the gut microbiota in comparison to individuals without the condition. This is corroborated by shifts in the gut microbiota across different levels of categorization. In fact, gut dysbiosis is postulated to be the instigating factor in the development of psoriasis among susceptible individuals. Several factors can influence gut dysbiosis including diet, genetic factors, sleep habits, physical activity, health status, medication use, and various environmental conditions. Evidence suggests that Th17 cells are vital for preserving the integrity of the intestinal epithelial barrier [27]. They can also contribute to the development of intestinal inflammation and the progression of autoimmune diseases outside the intestine by interacting with the microbiota, thereby serving as a link between host microbiota and immune-mediated inflammatory conditions. Psoriatic patients show a significant decrease in the abundance of SCFAs-producing bacteria such as *Prevotella*, *Akkermansia*, and *Ruminococcus* [27]. Lower levels of SCFAs, particularly butyrate, impair the suppression of Th17 cells and obstruct the differentiation of Tregs. The absence of butyrate, along with its anti-inflammatory properties, leads to a chronic, low-grade inflammatory state that weakens the gut barrier. This condition increases the risk of bacterial translocation, permitting bacterial DNA and other antigens to enter systemic circulation and trigger immune responses in distant areas, such as the skin. The gut microbiome in psoriatic patients is characterized by an abundance of bacteria that convert dietary carnitine and choline into trimethylamine. This trimethylamine is then metabolized by the liver into TMAO, a metabolite that promotes atherosclerosis and may elevate the risk of cardiovascular diseases in those with psoriasis [28].

Generally, the gut microbiome of patients with psoriasis shows an increase in *Actinobacteria* and *Firmicutes*, as well as an elevated *Firmicutes*-to-*Bacteroidetes* ratio (F/B ratio), all of which are linked to impaired gut epithelial barrier integrity. Similarly, the rise in the F/B ratio was also observed in patients with reduced physical activity and diseases such as IBD, obesity, type 2 diabetes, and several cardiovascular diseases [29]. Thus far, several studies have looked into gut dysbiosis in the human gut microbiome among patients suffering from psoriasis. Almost all of these studies have analyzed fecal samples, but they employed a range of molecular methods to assess microbial diversity. For instance, in a recent case-control study by Yunusbayev et al., fecal samples from 53 psoriasis patients and 47 healthy donors were subjected to deep sequencing to reconstruct the strain/species-level content of the gut microbiome. The study revealed no signs of reduced gut community diversity or significant structural differences between patients and healthy individuals. Nevertheless, there was a consistent yet subtle increase in certain bacteria among the patients, such as *Megasphaera elsdenii* and *Eubacterium* CAG 180. Furthermore, they demonstrated that these enriched species were associated with higher levels of biomarkers indicating intestinal and systemic inflammation, as well as liver function [30]. In another study from China, Wen et al. collected fecal samples from psoriasis patients (*n* = 32), healthy controls (*n* = 15), and their healthy partners (*n* = 17) for metagenomic shotgun sequencing analysis. Their findings revealed that the intestinal microbiota profiles of the psoriasis patients were markedly different from those of the healthy controls and their partners. Despite these differences, microbial diversity did not vary significantly across the three groups. At the phylum level, the relative abundances of *Firmicutes* and *Bacteroidetes* were found to be inversely correlated. Additionally, *Escherichia coli* was notably enriched in the psoriasis group compared to the healthy participants and their partners [31]. Likewise, Xia et al. utilized the same analytical approach to investigate fecal samples from psoriasis patients (*n* = 30) and a control group of healthy subjects (*n* = 15). Their study revealed that the gut microbiota in psoriasis patients showed a shift in the distribution of microbial taxa when compared to healthy individuals, although no significant differences in microbial diversity were observed [32].

**Fig. 2:**
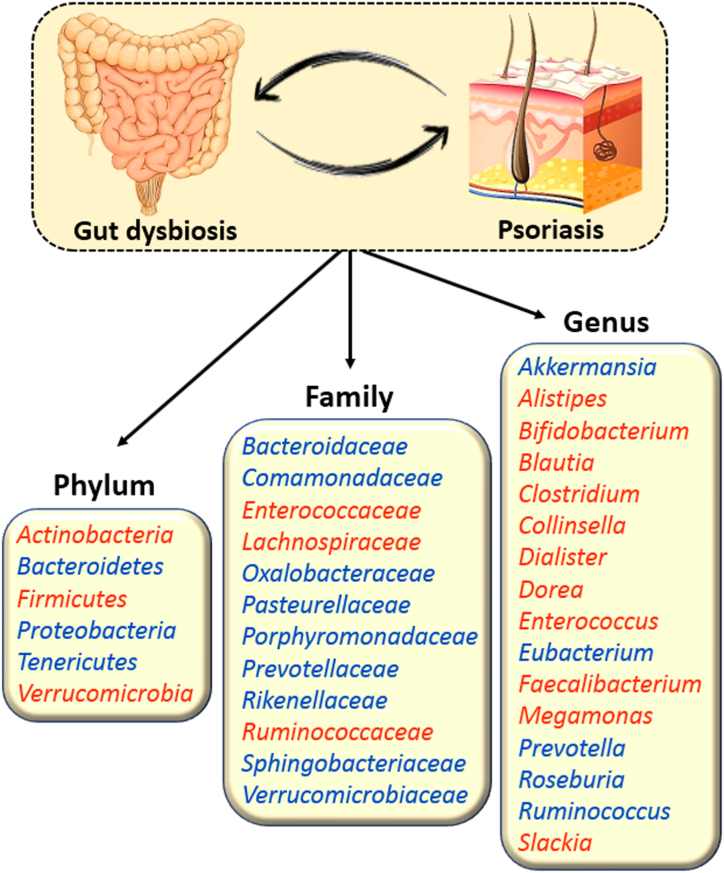
Changes in the hierarchical levels of phyla [[32], [33], [34], [35],[37], [38], [39], [40]], families [32,35,40], and genera [[31], [32], [33], [34],^37^,[39], [40], [41], [42], [43], [44]] of gut bacteria in patients diagnosed with psoriasis. Red color denotes an increase in population for family, genus, and phylum, whereas blue color denotes a decrease.

Other studies employed 16S ribosomal RNA (rRNA) sequencing for evaluating gut microbial diversity. Recently, a Brazilian study focused on the gut microbiome of adult males, comparing those with psoriasis (*n* = 21) to non-psoriasis controls, which included omnivores (*n* = 14) and vegetarians (*n* = 7). The study found that the F/B ratio was significantly higher in the psoriasis group than in the vegetarian group (*p* < 0.05). Additionally, the genera *Prevotella*, *Mogibacterium*, *Dorea*, *Bifidobacterium*, and *Coprococcus* were found to differ between psoriasis patients and vegetarians, while *Mogibacterium, Collinsella*, and *Desulfovibrio* were distinct from the omnivore group [33]. Likewise, in another study conducted in Spain, researchers explored the microbial diversity among psoriasis patients (*n* = 19) and a control group of healthy individuals (*n* = 20). The results indicated that the gut microbiota of those with psoriasis showed diminished diversity and a different relative abundance of certain bacterial taxa in contrast to the healthy controls [34]. In a comparable study conducted in Taiwan, Chen et al. employed the same approach to assess the diversity of the gut microbiota among psoriasis patients (*n* = 32) and a control group without psoriasis (*n* = 64). Their findings revealed a unique structure of fecal microbial communities in psoriasis patients, characterized by a higher prevalence of the phylum *Firmicutes* and a lower prevalence of the phylum *Bacteroidetes*. Notably, *Ruminococcus* and *Megasphaera*, both belonging to the phylum *Firmicutes*, were identified as the two genera with the most significant differences in abundance in psoriasis cases [35]. Another study from Italy also examined the gut microbiome profiles of psoriatic patients, comparing those who received anti-psoriatic systemic therapy (*n* = 10) with those who did not receive treatment (*n* = 20). Their findings revealed a decrease in bacterial biodiversity among the treated patients in contrast to their untreated counterparts. Additionally, significant differences in the relative abundances of *Akkermansia muciniphila* and *Bacteroides plebeius* were identified between the two groups [36]. Due to space constraints, we are unable to include all studies; therefore, only the most recent ones are presented. Fig. 2 illustrates the shifts that occur in the phyla, families, and genera of gut bacteria in individuals with psoriasis, based on the consensus results. Taken together, various limitations, including small sample size, differences in study population, methodologies, insufficient sequencing depth, possible contamination in the experimental process, and diverse factors affecting the microbial composition of feces, such as diet, contribute to inconsistencies in the results of these studies. Consequently, it is imperative to explore better methods to gain knowledge about the relationship between psoriasis and specific gut bacteria at the species level.

## 4 The bidirectional influence of psoriasis treatment and gut microbiome

Recent research has thrown light upon the connection between psoriasis treatments and alterations in the gut microbiome. For instance, narrow-band ultraviolet radiation B (NB-UVB) has been found to significantly impact gut microbiota by influencing the metabolism of vitamin D. One study examined the changes in gut microbiota among psoriasis patients receiving acitretin in combination with NB-UVB through 16S rRNA sequencing. A reduction in *Actinomyces*, *Prevotellaceae*, *Victivallis*, *Coprococcus*, and *Blautia* at the genus level was observed in the treated groups [45]. Another study noted a substantial enrichment of *Lactobacillus* and *Ruminococcus* genera in psoriasis patients treated with NB-UVB when compared to the healthy groups [46].

Biologics involve tumor necrosis factor‐alpha (TNF-α) inhibitors (adalimumab), IL-17A inhibitors (secukinumab and ixekizumab), IL-12/23 inhibitors (ustekinumab), and IL-23 inhibitors (guselkumab). According to one study, secukinumab treatment was associated with an increase in *Citrobacter* and a decrease in *Aeromonas*, *Bacteroides*, and *Ruminococcus* at the genus level. There was no substantial modification in the gut microbiome after ustekinumab therapy, except for a notable increase in the *Coprococcus* genus [47]. In a separate study, it was observed that various biologics led to a significant increase in the relative abundance of *Coprococcus* and *Adlercreutzia*, while causing a notable decrease in the relative abundance of *Dialister*, *Veillonella*, *Eggerthella*, and *Erysipelatoclostridium* in the bio-treated group as opposed to the non-bio-treated group. The bio-treated groups also showed a decrease in the F/B ratio within their gut microbiome [48].

Another field worth exploring is pharmacomicrobiomics, as findings have shown that gut microbiota can be utilized as a biomarker to predict therapeutic response. Manipulating the gut microbiota may raise the bioavailability and effectiveness of drugs, and hindering the enzymatic activity of certain bacteria could prevent them from metabolizing drugs into harmful compounds. The unique characteristics of an individual's microbiome could influence the clinical efficacy of certain drugs, reducing the likelihood of adverse reactions, as seen in conditions such as rheumatoid arthritis and ankylosing spondylitis [[49], [50], [51]].

Thus far, there are only a limited number of studies that have investigated the relationship between the gut microbiome and various therapies in patients with psoriasis. For instance, in a study carried out by Zhang et al. the gut microbiota changes in patients with ankylosing spondylitis and psoriatic arthritis (*n* = 20) were assessed (using a 16S rDNA sequencing approach) after treatment with anti-TNF-α (adalimumab), in comparison to healthy controls (*n* = 19). The findings indicated that *Bifidobacterium* and *Parasutterella* normalized following treatment, whereas *Escherichia*-*Shigella* and *Klebsiella* levels were reduced to normal values [52]. In another study, a total of 29 fecal samples from patients diagnosed with psoriatic arthritis/spondyloarthritis were analyzed both prior to and following treatment with a tumor necrosis factor inhibitor (TNFi; *n* = 15) or an anti-IL-17A monoclonal antibody inhibitor (IL-17i; *n* = 14). The sequencing methods employed included 16S rRNA, internal transcribed spacer (ITS), and shotgun metagenomics. Patients receiving IL-17i exhibited a higher abundance of *Clostridiales* and *Candida albicans* in comparison to those treated with TNFi [53]. Similarly, Valentine et al. evaluated the gut microbiome composition (using 16S rRNA sequencing) of psoriatic patients, focusing on those treated with biologic drugs versus those who were untreated. Their study comprised 10 individuals on biologic systemic therapy, including anti-TNF-α and anti-IL-12/23, and 20 patients who had not received any anti-psoriatic systemic therapies or topical corticosteroids. The analysis revealed a lower level of bacterial biodiversity among the treated patients in contrast to the untreated patients. Notable variations in the relative abundances of critical gut microbial communities, including *Akkermansia muciniphila* and *Bacteroides plebeius*, were identified between the two mentioned groups [36]. Overall, psoriatic patients who are treated with biologic drugs may exhibit notable differences in their gut microbiome composition when compared to untreated individuals. The lower levels of some potentially pathogenic species in those receiving treatment imply that biologic therapies might beneficially alter the gut microbiome. Moreover, a higher abundance of bacteria that contribute positively to the host's health may reflect the effectiveness of the therapy.

## 5 Oral probiotics for psoriasis treatment

Evidence suggests that probiotics could play a positive role in managing psoriasis. Preclinical studies have indicated that continuous use of oral probiotics can significantly improve the advancement of psoriasis and decrease the presence of inflammatory factors in animal models. According to one study, the oral administration of *Lactobacillus pentosus* GMNL-77 was found to have the potential to diminish erythematous scaling lesions in imiquimod-induced psoriasis-like skin inflammation in a BALB/c model. It also reduced the expression of TNF-α, IL-6, IL-23, IL-17A/F, and IL-22 mRNA and the population of Th17/Th22 cells [54]. Likewise, the oral administration of *Lactobacillus sakei* proBio-65 led to a decrease in skin lesions and pathological alterations, a reduction in skin thickness, and diminished levels of IL-19, IL-17A, and IL-23 mRNA expression [55]. Consistent with these findings, research revealed that the administration of *Escherichia coli* Nissle 1917 resulted in a decrease in skin lesions, skin thickness, and pathological changes. Moreover, it led to a decrease in serum IL-8, IL-17, IL-23, and TNF-α levels, and an increase in serum IL-10 levels. Additionally, there was a decrease in the expression of IL-17A, IL-17F, IL-23, and TNF-α mRNA, and an increase in the expression of IL-10 mRNA in the animal model [56].

The improvement of Psoriasis Area and Severity Index (PASI) scores and reductions in inflammatory markers such as C-reactive protein (CRP), IL-6, and TNF-α in patients have been observed in several randomized controlled trials (RCTs) investigating the effects of various probiotics including *Lactobacillus* and *Bifidobacterium* species, as compared to control groups. The PASI score is frequently employed in psoriasis research to grade the severity of psoriatic lesions and the patient's response to therapy. Typically, a PASI score between 5 and 10 indicates moderate disease, while a score exceeding 10 indicates severe psoriasis. A 75 % reduction in the PASI score is the current benchmark for most clinical trials in psoriasis and the criterion for efficacy of new psoriasis treatments approved by the US Food and Drug Administration (FDA) [57]. The efficacy of probiotic supplements in treating psoriasis is highlighted in Table 1, where the majority of clinical trials have shown favorable results. These include improvements in PASI score, reduction in proinflammatory cytokine levels, and enhanced quality of life for patients.

**Table 1:** Summary of the most relevant randomized controlled clinical trials investigating the efficacy of probiotic supplementation in patients with psoriasis.

Study group	Country	Intervention	Treatment duration	Key findings	Reference
Patients (*n* = 26) with mild to moderate chronic plaque psoriasis showing PASI <16 and healthy subjects (*n* = 22)	Ireland	Daily oral administration of *Bifidobacterium infantis* 35624 (sachets containing either 1 × 10^10^ CFUs or 5 g MLD as placebo)	8 weeks	• ↓CRP, ↓TNF-α, and ↓IL-6 levels in the probiotic group • ↓TNF-α and ↓IL-6 from LPS-stimulated PBMCs in healthy subjects in comparison to those receiving placebo	[58]
Patients (*n* = 90) with plaque psoriasis were randomized into two groups: probiotics (*n* = 45) and placebo (*n* = 45), all of whom received topical corticosteroid betamethasone in combination with CAL	Spain	Oral administration of a capsule (containing 1 × 10^9^ CFUs/capsule of *Bifiobacterium longum* CECT 7347, *Bifidobacterium animalis* subsp. *lactis* CECT 8145, and *Lactobacillus rhamnosus* CECT 8361 or MLD as placebo)	12 weeks	• ↓PASI in the probiotic group (30 of 45: 66.7 %) and the placebo group (18 of 43: 41.9 %) • Two patients did not complete the study • Modulation of the microbiota composition (↓*Micromonospora*, ↓*Rhodococcus*, ↑*Collinsella*, and ↑*Lactobacillus*) in the probiotic group • No differences in inflammatory marker levels (such as TNF-α, IFN-γ, IL-1β, IL-6, IL-12 and IL-23) between two groups were observed • No severe side effects were observed in the studied groups during the intervention	[59]
Patients (*n* = 50) with plaque psoriasis were randomized into two groups: probiotics (*n* = 25) and placebo (*n* = 25)	Iran	Twice-daily oral administration of a capsule (containing 1.8 × 10^9^ CFUs/capsule of *Lactobacillus acidophilus*, *Bifidobacterium bifidum*, *Bifidobacterium lactis*, and *Bifidobacterium longum* or MLD as placebo)	8 weeks	• ↓BDI-II, ↓DLQI, ↓PASI, and ↓PSS scores in the probiotic group in comparison to the placebo group • ↑TAC, ↓CRP, ↓IL-6, and ↓MDA levels in the probiotic group in comparison to the placebo group	[60]
Psoriasis patients (*n* = 27) received anti-psoriatic treatment	China	Oral administration of *Bacteroides fragilis* BF839	12 weeks	• ↓PASI in the probiotic group • One patient was excluded from the trial • Adverse effect: one case of constipation	[61]
Patients with mild to moderate psoriasis (*n* = 64) were randomized into two groups: treatment (*n* = 32) and placebo (*n* = 32)	Iran	Twice-daily oral administration of a Lactocare® capsule (containing 1 × 10^9^ CFU/capsule of *Lactobacillus casei*, *Lactobacillus acidophilus*, *Lactobacillus rhamnosus*, *Lactobacillus bulgaricus*, *Bifidobacterium breve*, *Bifidobacterium longum*, *Streptococcusthermophilus* with prebiotic FOS)	12 weeks	• ↑Serum levels of Fe, Zn, P, Mg, Ca, and Na • Eight patients from the intervention group and 18 patients from the control group discontinued the study due to the COVID-19	[62]

BDI: Beck depression inventory; CAL: Calcipotriol; CFU: Colony forming unit; COVID-19: Coronavirus disease 2019; CRP: C-reactive protein; DLQI: Dermatology Life Quality Index; FOS: Fructooligosaccharide; IFN-γ: Interferon-gamma; IL: Interleukin; LPS: Lipopolysaccharide; MDA: Malondialdehyde; MLD: Maltodextrin; PASI: Psoriasis Area and Severity Index; PBMCs: Peripheral blood mononuclear cells; TAC: Total anti-oxidant capacity; TNF-α: Tumor necrosis factor-alpha; ↑: Increased; ↓: Decreased.

## 6 Fecal microbiota transplantation (FMT)

FMT is the engraftment of intestinal microbiota from a healthy donor into a recipient's colon, aiming to restore the natural microbial community structure of the gut [63]. Currently, FMT has emerged as a successful treatment option for refractory and recurrent *Clostridium difficile* infections that do not respond to antibiotics. Since multiple major diseases are potentially connected to imbalances in the gut microbiome, FMT may have uses that extend beyond treating *C. difficile* infections. In recent years, FMT has emerged as a promising treatment approach for a range of diseases, including Crohn's disease, irritable bowel syndrome (IBS), graft-versus-host disease (GVHD), post-antibiotic diarrhea, constipation, encephalopathy, multiple sclerosis, autism, and metabolic syndrome, among others [63].

In one study, a patient diagnosed with plaque psoriasis and IBS received FMT treatments twice through endoscopy and colonoscopy. Following the intervention, improvements were noted in body surface area, PASI score, dermatology life quality index (DLQI), intestinal symptoms, and serum level of TNF-α, with no adverse reactions reported over the five-week period [64]. The available evidence regarding the efficacy of FMT in managing psoriasis is largely centered on patients with psoriatic arthritis. In this respect, FMT was found to be less effective in treating active peripheral psoriatic arthritis in an RCT, but no serious side effects were reported in patients with psoriasis [65]. A later study looked at how FMT affects inflammation-associated plasma proteins in patients with psoriatic arthritis. The results revealed that FMT had the most substantial positive effects on interferon-gamma (IFN-γ), Axin-1, and CCL25, and the most pronounced negative effects on CCL19 and IL-6 [66]. Further clinical trials are required to substantiate the observed advantages of FMT in managing disease severity among psoriatic patients, especially when utilized in conjunction with other treatment modalities.

## 7 Other potential and innovative therapies

Besides the therapies that have been mentioned, there are alternative therapeutic strategies that do not fit neatly into conventional categories. These approaches have the potential to help restore balance in the gut microbiota of psoriatic patients. One area worthy of research exploration is the oral administration of antibiotics in relation to changes in gut microbiota for treating psoriasis. The connection between antibiotics and psoriasis has been a topic of debate for many years [67]. While there is evidence pointing to the potential for antibiotics to improve, trigger, or worsen psoriasis, only a small number of studies have examined this relationship in depth. For example, the use of macrolides and rifampin has demonstrated improvement in the PASI scores for plaque-type psoriasis. On the other hand, there have been instances of exacerbation of generalized pustular psoriasis with amoxicillin, and plaque-type psoriasis with tetracyclines [67].

Significant progress has been achieved in microbiome engineering through the use of genetically modified bacteria or bacteriophages [68]. Historically, the techniques for engineering bacteria within the gut microbiome were limited to a few well-studied, easily culturable bacteria. Through the application of synthetic biology tools and approaches, microbiome engineering has primarily concentrated on several model organisms, particularly *Escherichia coli* and *Lactococcous lactis* [69]. Seminal works in the engineering of *L. lactis* to produce IL-10, IL-27, or certolizumab (anti-TNF-α) have also been demonstrated to improve colitis and Crohn's disease in mouse and rat models [[70], [71], [72]].

The increasing attention toward phage therapy has opened up new possibilities for using bacteriophages to combat a range of bacterial diseases. There is a growing body of evidence suggesting that phages are essential in the success of FMT therapy by rebalancing the dysbiotic gut microbiota. Fecal virome transplantation (FVT) is a modified version of FMT that involves filtering out the bacterial content from donor feces, helping to diminish the risk of bacterial infections. FVT have proven effective in managing various health issues, including obesity and type-2 diabetes, as well as preventing the onset of necrotizing enterocolitis [73]. Phages with lytic activity have been shown to successfully eradicated adherent-invasive *Escherichia coli* in a mouse model of colitis and human intestinal samples [74]. Moreover, they have been effectively employed to eliminate *Enterococcus faecalis* in a mouse model of ethanol-induced liver disease [[75], [76]]. Overall, further clinical trials with a larger number of participants are essential to validate the significance of these findings in humans and to investigate the efficacy of these potential therapeutic approaches for patients with psoriasis.

## 8 Concluding remarks

Dysbiotic changes in the gut microbiome composition may trigger immune responses that can ultimately lead to inflammatory diseases. Now that this connection has been established, researchers can delve into the complex relationships between commensal microorganisms and their hosts. Recent evidence has enriched our understanding of the bidirectional relationship between psoriasis and the gut microbiome. On the other hand, the presence of the skin's microbiome should not be underestimated, as it is essential for regulating the epidermis and influencing the body's immune system. Nevertheless, a detailed discussion of these topics is outside the scope of this review and will be deferred to a later publication. Unarguably, an interdisciplinary endeavor involving genomics, bioinformatics, clinical microbiology, and dermatology will be crucial for gaining a comprehensive understanding of the microbiome's influence on human health. To better understand the genomic aspects of the human microbiome and its potential role in psoriasis development, further research is necessary, including long-term studies that employ standardized sampling methods, suitable control groups, and thorough clinical data.

## CRediT authorship contribution statement

**Mojtaba Memariani:** Writing – review & editing, Writing – original draft, Visualization, Validation, Supervision, Project administration, Methodology, Investigation, Formal analysis, Conceptualization. **Hamed Memariani:** Writing – original draft, Software, Resources, Methodology, Investigation, Formal analysis, Data curation.

## Data statement

The datasets generated and/or analyzed during the current study are available from the corresponding author upon reasonable request.

## Data availability

Data will be made available on request.

## Funding

The authors declare that no funds, grants, or other support were received during the preparation of this manuscript.

## Declaration of competing interest

The authors declare that they have no known competing financial interests or personal relationships that could have appeared to influence the work reported in this paper.
